# Hemolysis scavenger proteins and renal function marker in children with sickle cell disease at steady state: A cross‐sectional study

**DOI:** 10.1002/hsr2.1177

**Published:** 2023-03-30

**Authors:** Fatima A. Fordjour, Alexander Kwarteng, Vivian Paintsil, Ernest Amanor, Ezekiel B. Ackah, Evans X. Amuzu, David S. Sackey, Alex Osei Yaw Akoto

**Affiliations:** ^1^ Department of Microbiology University for Development Studies Tamale Ghana; ^2^ Department of Biochemistry and Biotechnology, College of Science Kwame Nkrumah University of Science and Technology Kumasi Ghana; ^3^ Kumasi Centre for Collaborative Research in Tropical Medicine Kumasi Ghana; ^4^ Child Health Directorate Komfo Anokye Teaching Hospital Kumasi Ghana; ^5^ College of Health Sciences Kwame Nkrumah University of Science and Technology Kumasi Ghana; ^6^ School of Public Health Kwame Nkrumah University of Science and Technology Kumasi Ghana; ^7^ Haematology Unit Komfo Anokye Teaching Hospital Kumasi Ghana

**Keywords:** crisis, hematolgy, hemolysis, proteins, renal/kidney, scavenger, sickle cell

## Abstract

**Background and Aims:**

Hemolysis is a fundamental feature of sickle cell disease (SCD) contributing to the vaso‐occlusive crisis of patients. The objectives of the study were to assess the link between hemolysis proteins and hematological parameters, and to validate cystatin C (CYS C) as a potent renal marker in diagnoising SCD.

**Method:**

Here, a cross‐sectional study carried out at the pediatric SCD clinic of the Komfo Anokye Teaching Hospital comprised 90 SCD children (HbSC, HbSF, and HbSS). ANOVA, *t*‐test, and Spearman's rank correlation analysis were done. Elevated proteins levels were compared to standard values; alpha‐1 microglobulin (A1M) (1.8−65 µg/L), CYS C (0.1−4.5 µmol/L), and haemopexin (HPX) (500−1500 µg/mL).

**Results:**

The mean (standard deviation) age of participants was 9.830 (±0.3217) years, and 46% of them were males. From simple descriptive analysis, we observed that all but one patient had their HPX level below the reference range (<500 µg/mL). Here, A1M levels were shown to be within the appropriate reference range for all the patients except few patients. CYS C levels were also all within the required reference values. A Spearman's rank correlation test between full blood count and HPX generally suggested a weak but positive correlation; RBC (coef. = 0.2448; *p* = 0.0248), HGB (coef. = 0.2310; *p* = 0.030), hematocrit (coef. = 0.2509; *p* = 0.020), and platelet (coef. = 0.1545; *p* = 0.160). Mean corpuscular volume (coef. = ‐0.5645; *p* = 0.610) had a stronger but negative correlation with HPX. This study depicts a positive and stronger association between CYS C and HPX levels (coef. = 0.9996; *p* < 0.0001), validating the use of CYS C as a useful marker of renal function in persons with SCDs.

**Conclusion:**

In the present study, we show that A1M levels were normal for most of the patients, hence CYS C levels are not alarming in this study. Further, there exists a correlation between hemolysis scavenger proteins and hematological parameters.

## 1 INTRODUCTION

Sickle cell disease (SCD) is a genetic form of anemia in which mutated forms of hemoglobin distort the red blood cells into crescent shapes at low oxygen levels. About 300,000 babies are born each year with sickle cell anemia, with 75% of this population in Africa.^1^ In every 5 people with SCD, 1 develops renal abnormalities which could lead to chronic kidney disease (CKD).^2^ There are several variants of SCD: Sickle cell anemia (HbSS), sickle hemoglobin‐C (HbSC), and sickle faetal hemoglobin (HbSF). The HbSC form of SCD is mostly common in Africa.^3^ In general, patients with the HbSC form of the disease have milder abnormalities than their homozygous (HbSS) counterparts due to less anemia.^4^, ^5^ Delays in diagnosis of SCD may increase the risk for developing CKD eventually leading to increased morbidity and mortality.^6^

Although renal complications in sickle cell remains painless and develops gradually over time, its effect results in organ dysfunction. As a vaso‐occlusive disease, the contribution of anemia and hemolysis to the progression of both acute and chronic sickle cell complications cannot be underestimated.^7^, ^8^, ^9^ Anemia is a common presentation in SCD; resulting mainly from hemolysis, and is a reflection of disease severity.^9^, ^10^

Intravascular hemolysis in SCD is marked by the scavenging activities of danger associated molecular patterns (DAMPs) (i.e., haptoglobin [HPG], haemopexin [HPX], and alpha‐1 microglobulin [A1M]).^7^, ^9^ HPG and HPX neutralize the DAMPs through the liver, with A1M stepping in only when HPX becomes depleted. Hemolytic anemia in SCD may also increase with malaria infection.^11^ Despite the detrimental contribution of anemia and hemolysis to organ damage, there is no hematological technology to determine marked injury. The aforementioned scenarios suggest that levels of the hemolysis scavenger proteins (HSPs) could be the only indication of intravascular hemolysis.^12^ Hemolysis due to reduced/depleted HPX, and high levels of A1M has been linked to poor kidney function in sickle mice studies, and further stated that, this incident coupling with anemia may also lead to kidney dysfunction and damage.^8^, ^12^

The serum levels of urea and creatinine are commonly used as renal function tests. This notwithstanding, studies have shown that serum levels of cystatin C (CYS C) are a more precise test of renal function (as represented by the glomerular filtration rate, GFR) than urea and creatinine.^13^, ^14^, ^15^ A study on a cohort of pediatric SCD patients in Ghana rather used urea and creatinine to assess kidney function.^12^ Thus, previous study conducted on organ damage in SCD (ORDISS), may not be able to detect mild renal impairment, and predict risk of developing CKD, as the serum levels of urea and creatinine are inaccurate at detecting this complication. Thus, there is the need to study the effect of SCD on renal function using a biomolecule whose serum levels provides a precise understanding on the function of the kidneys, especially, at the early stages of renal complication. Furthermore, studies that have reported on anemia and hemolysis in relation to kidney function and other organ functions in SCD have mostly involved mouse models and/or SCD patients ^8^, ^16^, ^17^ outside of Ghana; hence, there is minimal data, if any, on Ghanaian SCD patients. This warrants the need to provide such laboratory data on HSPs in relation to kidney function in this cohort. Provision of such data and knowledge from this study will contribute to improving the management of SCD patients, and as well serve as a pedestal for further studies on the laboratory presentations of pediatric SCD patients at steady state. Here, we showed that hemolysis is mainly followed by depletion in HPX levels and a rise in A1M levels. Furthermore, we also observed a link between HPX and CYS levels validating CYS C as a useful renal marker.

## 2 MATERIALS AND METHODS

### 2.1 Study design

This was a cross‐sectional study carried out from July 2021 to November 2021 at the pediatric SCD clinic at the Komfo Anokye Teaching Hospital (KATH). The study comprised 90 children with SCD. After explaining the study procedures and obtaining informed consent from participants, screening questionnaires and case report forms were administered to collect data (age, sex, sickle status, malaria, FBC, and HSPs measurement). The population were children with confirmed SCDs attending clinic visits at KATH. Children with SCD of all sexes, in a steady‐state and from the ages of 5−14 years were recruited and included in the study. Steady‐state was defined as the absence of clinical symptoms or steady‐state clinically confirmed by a physician.

### 2.2 Sample size estimation and sampling technique

The minimum sample size estimated for the study was 90. This was determined using the Fisher's formula for sample size calculation, N = [z^2^p(1−p)]/d,^2^ ^18^ using a reported prevalence of 6%^6^ of steady‐state children with SCD. Specifically, N was the minimum sample size estimated; z was the point of standard normal distribution curve which was set at 1.96 (95% confidence interval); p was the assumed prevalence rate; and d was the degree of precision which was set at 6%. This study employed a random sampling technique in recruiting study participants. The registry of the sickle cell clinic was reviewed for selection of all participants.

### 2.3 Data collection

Caregivers and/or study participants were interviewed with an electronic semistructured questionnaire hosted on the School of Medicine and Dentistry, KNUST, Research Electronic Data Capture (REDCap) server.^19^ REDCap is a secure web application for building and managing online surveys and databases. To ensure that quality data was collected, the questionnaires were mainly answered by caregivers on behalf of study participants below the ages of 13 years whilst participants from 13 years and above responded to questionnaire and in some cases were assisted by their caregivers. Moreover, questions were interpreted in the local language where necessary for easy comprehension. Medical records were reviewed for each study participant for the clinic visit to confirm their steady state and complete other relevant variables. The questionnaire was categorized into demographic data, clinical characteristics, and medical history. Furthermore, the laboratory results of study participants were entered into a laboratory documentation sheet hosted on REDCap.

### 2.4 Study procedure

Selected patients were screened, and eligible ones were enrolled in the study. Permission was sought from parents and guardians of children who became eligible by signing/thumb printing an informed consent form (5−7) or assent (8−14 years) if needed. About 5 mL (2 mL EDTA and 3 mL serum) of blood samples were taken from each participant. Samples were transported on ice to a laboratory within 2−4 h for hematological assessment. Control samples were analyzed before assessing the parameters of the samples. The remaining samples (both serum and EDTA) were centrifuged and stored at minus 20°C freezer pending batch ELISA analysis (HPX, A1M, and CYS C). ELISA was performed at the Central Research Laboratory of KNUST according to the manufacturer's (Shanghai Chemical Ltd) protocol. The spectrophotometer used for reading the samples was calibrated for accurateness before the procedures.

### 2.5 Statistical analysis

Raw data was entered onto Microsoft Excel and statistical analysis was done using Micorsoft Excel and GraphPad Prism version 5.0. Descriptive statistics were performed for participant demographics, clinical, and laboratory characteristics. Categorical variables were expressed as frequency/percentage while continuous variables were expressed as mean/standard deviation at 95% confidence interval according to the distribution pattern of the variables. A comparison of numerical variables was analyzed using Mann−Whitney and Kruskal−Walli's tests in GraphPad Prism. Two‐way ANOVA was also performed on quantitative variables. A graphical presentation of numerical and categorical variables was performed. The relationship between continuous variables was determined by Spearman's rank test for correlation. Statistical significance was considered at *p* < 0.05.

## 3 RESULTS

### 3.1 Demographics of study participants

A total of 90 participants with SCD were recruited into this study (HbSS, *n* = 56), (HbSC, *n* = 25), and (HbSF, *n* = 9). The age of participants ranged from 5 to 14 years with a mean age of 9.830 (±3.018) years. There was a significance difference in ages (*p* < 0.001). Moreover, among the patients, HbSC (*p* < 0.010) and HbSS (*p* < 0.010) were significantly older in years than HbSF patients. We observed that 80% of the patients were on hydroxyurea treatment, with a higher proportion being HbSS variant as shown in Table 1. The full blood count parameters were compared with their respective reference values (Table 2).

**Table 1:** General characteristics of study participants (*n* = 90).

Variable	Frequency	Percentage
Gender		
Female	44	48.9 (44/90)
Male	46	51.1 (46/90)
Age (years)		
5−10	52	59.1 (52/88)
11−14	36	40.9 (36/88)
Mean ± SD	9.830 ± 3.018	
SCD genotype		
HbSC	25	27.8 (25/90)
HbSF	9	10 (9/90)
HbSS	56	62.2 (56/90)
Patients on hydroxyurea		
Yes	67	74.4 (67/90)
No	23	25.6 (23/90)
SCD genotype on hydroxyurea		
HbSC	7	10.4 (7/67)
HbSF	9	13.4 (9/67)
HbSS	56	83.5 (56/67)

Abbreviation: SCD, sickle cell disease.

**Table 2:** Comparison of hematological values to reference values among SCD patients.

FBC/reference range	HbSC (mean ± SD)	HbSF (mean ± SD)	HbSS (mean ± SD)	*p* Values
RBC (2.5−5.50)	3.878 ± 0.8197	2.894 ± 0.5588	2.818 ± 1.315	0.0001
HGB (8.0−17.0)	9.796 ± 1.296	8.856 ± 1.081	8.020 ± 1.288	0.0001
HCT (26.0−50.0)	27.29 ± 3.717	24.40 ± 2.958	22.32 ± 3.394	0.0001
MCV (86−110.0)	71.96 ± 8.876	85.68 ± 10.49	86.11 ± 10.84	0.0001
PLT (50−400)	299.5 ± 106.6	345.4 ± 206.8	311.9 ± 154.6	0.9960
WBC (3.00−15.00)	9.412 ± 3.463	7.199 ± 3.116	10.14 ± 3.565	0.0467

Abbreviations: HCT, hematocrit; HGB, hemoglobin; MCV, mean corpuscular volume; PLT, platelet; RBC, red blood cell; SCD, sickle cell disease; WBC, white blood count.

Mean ± SD values of scavenger protein among variants of SCD compared to their respective reference values.

*p* Values generated by Kruskal−Wallis test.

### 3.2 Description of hematological parameters among SCD patients in steady state

To assess the occurrence of anemia among the SCD variants, we compared RBC, HGB, hematocrit (HCT), mean corpuscular volume (MCV), platelet (PLT), and white blood count (WBC) values to their respective reference range (Table 2). The mean (standard deviation) RBCs (2.818 ± 1.315), HGB (8.020 ± 1.288), and HCT (22.32 ± 3.394) were least recorded in the HbSS variant as compared to the HbSC (RBC = 3.878 ± 0.8197, HGB = 9.796 ± 1.296, HCT = 27.29 ± 3.717) and HbSF (RBC = 2.894 ± 0.5588, HGB = 8.856 ± 1.081, HCT = 24.40 ± 2.958) variants. On the other hand, the highest mean (standard deviation) MCV and WBCs (86.11 ± 10.84 and 10.14 ± 3.565, respectively) were seen in the HbSS genotype, with the least recorded in the HbSC variant. High platelet count is normally seen in SCD patients at steady state, here we measured the highest mean PLT count in the HbSF (345.4 ± 206.8), followed by HbSS (311.9 ± 154.6) and the least in the HbSC variant (299.5 ± 106.6).

### 3.3 Hematological parameters reflect degree of hemolysis in SCD at steady state

To establish a link between the threshold of hemolysis and the blood count indices (RBC, HGB, HCT, MCV, PLT, and WBC), a Spearman's rank correlation test was performed. It was observed that RBC (coef. = 0.2448), HGB (coef. = 0.2310), HCT (coef. = 0.2509), PLT (coef. = 0.15450), WBC (coef. = 0.09444), and LYMPH (coef. = 0.00012) had a weak but positive correlation with HPX. Albeit MCV (coef. = −0.5645) had a stronger but negative correlation with HPX levels (Table 3).

**Table 3:** Correlation of full blood count parameters and proteins (A1M, CYS C, and HPX).

Variables	A1M (Spearman's *r*: *p* value)	CYS (Spearman's *r*: *p* value)	HPX (Spearman's *r*: *p* value)
RBC	0.2484: 0.0227	0.2471: 0.0234	0.2448: 0.0248
HGB	0.2340: 0.0322	0.2313: 0.0343	0.2310: 0.0345
HCT	0.2537: 0.0199	0.2514: 0.0211	0.2509: 0.0213
MCV	−0.06027: 0.5860	−0.05883: 05950	−0.5645: 0.6100
PLT	0.01567: 0.1545	0.1603: 0.1451	0.1545: 0.1604
WBC	−0.009689: 0.3806	−0.09226: 0.4039	0.09444: 0.3928

Abbreviations: A1M, alpha‐1 microglobulin; CYS C, cystatin C; HCT, hematocrit; HPX, haemopexin; MCV, mean corpuscular volume; PLT, platelet; WBC, white blood count.

Spearman's rank correlation test.

To manage SCD properly, hemolysis must be closely monitored. A1M, the second haeme scavenger, was subjected to a Spearman's rank correlation test and we observed that RBC (coef. = 0.2484), HGB (coef. = 0.2340), HCT (coef. = 0.2537), and PLT (coef. = 0.1567) had weak but positive correlation with A1M, nonetheless, MCV (coef. = −0.006027), WBC (coef. = −0.009689), and LYMPH (coef. = −0.003312) had a weak but negative correlation with A1M (Table 3).

### 3.4 Variations in red cell indices: An indicator of crisis among SCD variants at steady state

**Figure 1:**
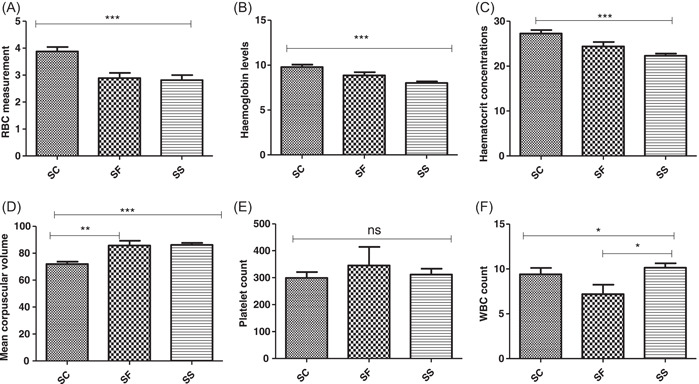
(A) Variations of RBC count among the variants of SCD. (B) Variations of HGB levels among the variants of SCD. (C) Levels of HCT count among variants of SCD. (D) Levels of MCV among SCD patients. (E) Levels of PLT count among variants of SCD. (F) Levels of WBC count among variants of SCD, where *p* < 0.01*, *p* < 0.001,** and *p* < 0.0001*** generated by Kruskal−Wallis test. HCT, hematocrit; MCV, mean corpuscular volume; PLT, platelet; SCD, sickle cell disease; WBC, white blood count.

The alteration in red cell indices is an indicator of anemia in SCD (commonest pathophysiology of SCD). This section recorded the differences in red cell indices among SCD variants. A Kruskal−Walli's test showed a significant difference among the SCD genotypes for the red cell indices (RBC, HGB, and HCT), with the highest counts in the HbSC variants, followed by HbSF and the least in the HbSS variant (Figure 1A−C). Low levels of red cells below reference points are critical signs of anemia and possible crisis in SCD. Here, we estimated the proportion of patients whose red cell parameters were below, within or above the reference values (Table 4).

**Table 4:** Comparison of hematological values of SCD patients to reference values.

FBC/reference	Values below reference values	Values within reference values	Values above reference values
RBC (2.5−5.5)	*N* = 25 (SC = 2; SF = 3; SS = 20)	*N* = 57 (SC = 21; SF = 6; SS = 30)	*N* = 2 (SC = 1, SS = 1)
HGB (8.0−17)	*N* = 29 (SC = 2; SF = 2; SS = 25)	*N* = 55 (SC = 22; SF = 7; SS = 26)	‐
HCT (26.0−50.0)	*N* = 58 (SC = 8; SF = 6; SS = 44)	*N* = 26 (SC = 16; SF = 3; SS = 7)	‐
MCV (86−110.0)	*N* = 55 (SC = 22; SF = 5; SS = 28)	*N* = 29 (SC = 2; SF = 4; SS = 23)	‐
PLT (50−400)	‐	*N* = 69 (SC = 20; SF = 7; SS = 42)	*N* = 15 (SC = 4; SF = 2; SS = 9)
WBC (3.0−15.0)	*N* = 1 (SF = 1)	*N* = 78 (SC = 23; SF = 8; SS = 47)	*N* = 5 (SC = 1; SS = 5)

Abbreviations: HCT, hematocrit; MCV, mean corpuscular volume; PLT, platelet; SCD, sickle cell disease; WBC, white blood count.

Number of SCD patients presenting with FBC parameters below, within and above their respective reference values, *SC = HbSC; SF = HbSF; SS = HbSS.

### 3.5 Low mean corpuscular volume predicts iron deficiency among SCD at steady state

High mean corpuscular volume is an indicator of iron stores in SCD patients, especially after hydroxyurea treatment, however, we observed low MCV in a higher proportion of our patients. In Table 2, the highest mean MCV was recorded in the HbSS variant and the least in the HbSC variant. A stronger association was seen between MCV and HPX, depicting possible hemolysis in some of our patients (Table 3).

### 3.6 Expression of scavenging proteins predicts degree of hemolysis

**Figure 2:**
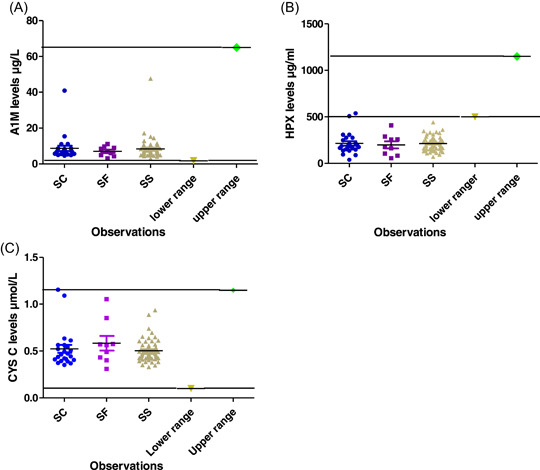
(A) Comparison of A1M levels to standard reference values. (B) Comparison of HPX levels to standard reference values. (C) Comparing Cystatin C levels to standard reference points. A1M, alpha‐1 microglobulin; HPX, haemopexin.

Levels of proteins that scavenge free haeme in the cell predicts the occurrence of hemolysis/crisis. To establish whether patients had haemolys in this study, we compared levels of scavenger proteins to their respective standard values (HPX [500−1500 µg/mL] and A1M [1.8−65 µg/L]). The mean (standard deviation) hemolysis proteins were 353.5 ± 741.8 and 10.60 ± 18.25 for HPX and A1M, respectively. Simple descriptive analysis showed that HPX (main haeme scavenger) levels were depleted in all but 1 of the SCD patients, indicating low levels of HPX as shown in Figure 2B. Elevated A1M (the second haeme scavenger) denotes depleted/overwhelmed HPX. Although A1M levels were elevated, most of the patients had their A1M levels within the appropriate reference values (Figure 2A).

Further, gender and age comparative analysis showed no statistic significant however, females and the older age group (11−14 years) had higher scavenger proteins (HPX and A1M) than their respective counterparts (males and 5−10 age group).

### 3.7 Levels of CYS C marker is an indicator of renal nephropathy in SCD at steady state

Early detection of renal complications is crucial in the management of SCD, here we assessed the levels of CYS C protein marker in our SCD cohort by comparing their protein values to reference values of 0.1−4.5 µmol/L. The mean (standard deviation) CYS C value was 0.8018 ± 1.645. Kruskal−Wallis' test and descriptive analysis showed that, CYS C levels were within the appropriate reference range for all, but one patient (Figure 2C). The HbSC variant had the highest CYS C levels. Gender and age comparative analysis also showed no significant difference, nonetheless, females and the older age group (11−14 years) had higher levels of CYS C than their respective counterparts (males and 5−10 age group).

### 3.8 Interdependence of HSP and renal function marker in SCD

**Figure 3:**
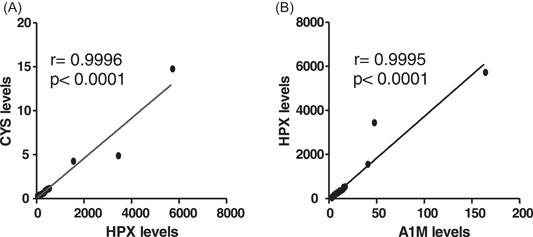
(A) Correlation of CYS C levels and A1M levels could be an indicator of renal dysfuncton. (B) Correlation test of A1M and HPX denoting the scavenging ability of A1M after HPX is depleted/overwhelmed, where *r = 1 indicates perfect association between CYS C and A1M; A1M and HPX and *p < 0.0001 denotes statistical significance. A1M, alpha‐1 microglobulin; CYS C, cystatin C; HPX, haemopexin.

This part establishes an association between HPX and CYS, both of which are known to measure specific liver and kidney activities, respectively. From Figure 3A, there was a positive and stronger association between CYS C and HPX levels (coef. = 0.9996; *p* < 0.001). This shows that CYS C level is dependent on HPX levels. Figure 3B also shows a stronger and positive correlation between A1M levels and HPX levels (coef. = 0.9995; *p* < 0.001).

## 4 DISCUSSION

This study was mainly to evaluate the levels of HSPs and to validate the use of CYS C as a useful renal marker in persons with SCD. Here, we established a link between hematological parameters and hemolysis proteins among children living with sickle cell in Ghana. It is crucial for people with blood disorders such as SCD to track their overall health. In this study, HbSS recorded the highest WBC and PLT counts and this supports findings that there is elevation of WBC and PLT count in the homozygous variant of the sickle hemoglobin diseases.^20^, ^21^ Variation in WBC and PLT counts could be accompanied by mild to severe hemolysis,^22^ one of the characteristic features in the pathophysiology of SCD.^23^ SCDs mainly affect red blood cells but can sometimes modify platelets and white blood cells.^24^ People with SCD generally have anemia, (fewer red blood cells than normal) as sickled red blood cells do not circulate in the blood long enough as normal ones do, leading to lower hemoglobin levels.^24^

A recent report from our laboratory shows that successive blood transfusion during anemia is usually accompanied by iron overload in SCD.^25^ In SCD, there is an increased tendency for RBC lysis and adhesion, thereby leading to hemolysis. A Positive association was observed between HPX and A1M as shown in Table 3, however, this association was weak. The extent of hemolysis is driven by RBC instability and the extent of potential heme toxicity by changes in heme that affect it's binding to globin.^26^ Chronic hemolysis usually seen in SCD shorten red cell survival as well as low erythropoietin in SCD, reducing levels of HGB and HCT as observed in our present study.^21^ The findings in this study are in accordance with other studies in Africa and other low‐income and middle‐income countries that showed significant impairment in RBC and other hematological indices.^26^ Indeed, we have shown that total red RBC count, hemoglobin (HGB) concentration, HCT count, and MCV were substantially reduced in almost 65% of patients from this study.^26^, ^27^ Interestingly, low levels of MCV as seen here in a higher proportion of patients could reflect low compliance of hydroxyurea treatment in patients from this study as some reported of breakages in treatment.

Free heme drives endothelial cell expression of adhesion molecules to which platelets and RBCs attach, ultimately blocking blood flow.^28^ Here, we observed a total decline in HPX levels in almost all SCD patients except one. The downregulation of HPX (the scavenger protein of cell free haeme) is an indication of hemolysis in SCD. There were no statistical differences among the SCD cohort on gender and age comparison, nonetheless higher levels of HPX were seen in the females and the older age group. This supports findings that there is no statistic differences in HPX levels between males and females.^28^, ^29^ This scenario buttresses our observation of HPX depletion in almost all SCD cohort in this study, confirming HPX levels an indicator of hemolysis in hemolytic diseases such as SCD. Studies have also shown that adult levels of HPX are attained 6 months after birth,^17^ and thus, confirms that adult levels of HPX were already attained in all study participants preceding our study (age criteria ranged from 4 to 14 years).

The main role of A1M is to take over the detoxification activity of free haeme through the kidney when HPX is depleted.^8^, ^28^ Therefore, low levels of HPX but high A1M levels denote hemolysis. From this study, we observed that as HPX levels get depleted, A1M levels were gradually rising, however, levels were considerably within the appropriate reference range.^28^ Mouse model study has reported that excess heme is usually directed to the liver in healthy mice while excess heme travels to the kidney in SCD mice leading to chronic kidney damage.^30^ In the present study, our data indicate that as HPX levels get depleted, A1M levels rises gradually, denoting a positive and stronger correlation between HPX and A1M. Elsewhere, it has been reported that A1M levels are increased 1.6‐folds in SCD as compared to healthy controls.^31^, ^32^ This further confirms what has been observed in this study with A1M levels increasing averagely in HbSS variant than the other variants. This study further corroborates studies reporting that detoxification of free heme during hemolysis in SCD is handled by A1M through the kidney.^30^, ^31^ Wester‐Rosenlöf et al. suggested A1M to be an endogenous scavenger of heme and radicals, as treatment of A1M in animal model resulted in protection of organs from the side effect of depleted hemolytic markers such as HPX.^33^

The commonest renal diagnostic approach for estimating GFR is creatinine. Due to its lack of sensitivity for early detection of CKD, it has become important to find other markers that are potent in diagnosing kidney dysfunction earlier especially in SCD.^34^, ^35^, ^36^ Studies utilizing CYS C as a renal functional marker in SCD are few, however, reports indicates they are sensitive than other renal markers.^13^, ^37^ A study using 20 cohort of SCD children established a correlation between CYS C and albuminuria,^38^ and later found CYS C to be more sensitive.^13^ Here, we showed that most of our patients' CYS C levels were within reference range. This observation could be as a result of 80% of our patients been on hydroxyurea; a drug known to reduce renal nephropathy. Several studies have confirmed the ability of hydroxyurea in reducing hemolysis in SCD,^13^, ^15^, ^35^ hence the drug can regulate CYS C level which is known to be a potent marker of kidney function especially in hemolytic diseases.

From this study, 80% of the SCD patients were on hydroxyurea confirming their steady state. It was observed that patients who disclosed intermittent breakages in the medication suffered crisis until they resumed medication. This corroborates a study conducted in the 1990s on adults using hydroxyurea, and this has given insight for other studies that had significant data with the treatment of pediatric SCD on hydroxyurea.^39^, ^40^, ^41^ One such study was the REACH trial in sub‐Saharan Africa which shown tremendous improvement with about 90% success rate after treatment with hydroxyurea.^42^ Though hydroxyurea is an established treatment in children and adults with SCD, data on safety in Africa is limited, where coexisting conditions and malnutrition could impact toxicity thresholds. Additionally, more data is needed to determine if the high adherence can be maintained with less monitoring and to observe for any long‐term adverse effects. The efficacy of hydroxyurea is noteworthy and therefore warrants further studies to explore the relationship between the drug and other possible related inflammatory markers that could influence nephropathy.

Children with SCD in sub‐Saharan Africa are at risk of malaria morbidity and mortality.^43^ This study aimed to evaluate the influence of malaria on hemolysis in SCD. Another interesting part of this study is that none of the study participants, but one had plasmodium infection. Studies have shown that the presence of heme and sickle red blood cells confers protection during malaria infection.^43^, ^44^ Another factor may be due to the extensive education given to caregivers of SCD patients (children) in the present study to always sleep under insecticide treated nets, which is very necessary for SCD management in Africa.

This study has provided baseline information that can be used in the management of SCD patients especially in Ghana. Nevertheless, a key limitation of this study is that our study participants were all in their steady state of the disease which does not reflect the extent of danger SCD patients usually experience when in crisis, therefore making it impossible to make a comparison based on the different states of the disease. Another limitation is that healthy controls should have been recruited into the study to compare levels of hemaolysis scavenger protein, as available data on such comparison can only be found in mice with SCD. Larger population with both healthy controls and SCD patients should be conducted to extensively study these proteins (HPX and A1M) and their interactions with other factors.

## 5 CONCLUSION

The present study has shown that HPX levels are strongly associated with A1M and therefore, as HPX gets depleted, A1M gradually rises leading to kidney dysfunction. We showed that, most of the hematological indices fall below reference points in SCD. Moreover, there is variations in levels of HSPs (HPX and A1M) and renal function marker (CYS C) in the variants of SCD (HbSC, HbSF, and HbSS).

## AUTHOR CONTRIBUTIONS

**Fatima A. Fordjour**: Conceptualization; data curation; formal analysis; funding acquisition; investigation; methodology; project administration; resources; software; validation; visualization; writing—original draft; writing—review & editing. **Alexander Kwarteng**: Conceptualization; supervision; writing—review & editing. **Vivian Paintsil**: Funding acquisition; supervision; visualization; writing—review & editing. **Ernest Amanor**: Methodology; software. **Ezekiel B. Ackah**: Conceptualization; methodology. **Evans X. Amuzu**: Resources; software. **David S. Sackey**: Data curation; methodology. **Alex Osei Yaw Akoto**: Funding acquisition; resources; supervision.

## CONFLICT OF INTEREST STATEMENT

The authors declare no conflict of interest.

## ETHICS STATEMENT

Ethical clearance was obtained from the KATH Institutional Review Board (IRB) with reference: KATH IRB/CA/064/21. The background, aims, and study procedures were thoroughly explained to caregivers and patients. Consent and assent were obtained before any study participant was enrolled onto the study. Any study‐related procedure/documentation was completed in compliance with the appropriate regulatory authorities. Recruitment and sampling procedures was conducted in accordance with the WHO guidelines for good laboratory practice.

## TRANSPARENCY STATEMENT

The lead author Fatima A. Fordjour affirms that this manuscript is an honest, accurate, and transparent account of the study being reported; that no important aspects of the study have been omitted; and that any discrepancies from the study as planned (and, if relevant, registered) have been explained.

## Data Availability

The data and materials are available in the corresponding author's institution and will be made available upon formal request.
